# Current strategies for mutation detection in phenotype-driven screens utilising next generation sequencing

**DOI:** 10.1007/s00335-015-9603-x

**Published:** 2015-10-08

**Authors:** Michelle M. Simon, Eva Marie Y. Moresco, Katherine R. Bull, Saumya Kumar, Ann-Marie Mallon, Bruce Beutler, Paul K. Potter

**Affiliations:** Medical Research Council Harwell (Mammalian Genetics Unit and Mary Lyon Centre), Harwell Campus, Oxfordshire, OX11 0RD UK; Center for the Genetics of Host Defense, University of Texas Southwestern Medical Center, Dallas, TX 75390 USA; Nuffield Department of Medicine and Wellcome Trust Centre for Human Genetics, Oxford University, Oxford, UK; MRC Human Immunology Unit, Weatherall Institute of Molecular Medicine, Oxford, UK

## Abstract

Mutagenesis-based screens in mice are a powerful discovery platform to identify novel genes or gene functions associated with disease phenotypes. An *N*-ethyl-*N*-nitrosourea (ENU) mutagenesis screen induces single nucleotide variants randomly in the mouse genome. Subsequent phenotyping of mutant and wildtype mice enables the identification of mutated pathways resulting in phenotypes associated with a particular ENU lesion. This unbiased approach to gene discovery conducts the phenotyping with no prior knowledge of the functional mutations. Before the advent of affordable next generation sequencing (NGS), ENU variant identification was a limiting step in gene characterization, akin to ‘finding a needle in a haystack’. The emergence of a reliable reference genome alongside advances in NGS has propelled ENU mutation discovery from an arduous, time-consuming exercise to an effective and rapid form of mutation discovery. This has permitted large mouse facilities worldwide to use ENU for novel mutation discovery in a high-throughput manner, helping to accelerate basic science at the mechanistic level. Here, we describe three different strategies used to identify ENU variants from NGS data and some of the subsequent steps for mutation characterisation.

## Introduction

Forward genetic screens have been successful in identifying and functionally characterising hundreds of disease-related genes in mice (Acevedo-Arozena et al. 2008; Bull et al. 2013; Potter et al. 2015; Wang et al. 2015). This approach typically uses a DNA damaging agent such as *N*-ethyl-*N*-nitrosourea (ENU) to mutagenize male (G_0_) mice thus inducing random point mutations throughout the germline. Subsequent phenotyping screens on the progeny of these mice are used to identify mice with phenotypes that can mimic human disease and highlight key pathways. The random nature of this approach (no particular gene is targeted) means that novel causative genes can be discovered with no prior annotation required. The mouse is 99 % homologous to humans making it an ideal model organism to study human disease (Mouse Genome Sequencing et al. 2002). The mouse reference—C57BL/6J—was originally sequenced in 2001; since then multiple updates to the assembly have rendered the reference a stable and reliable background to identify sequence variations (Church et al. 2009). This was and is imperative to identifying ENU mutations because detection traditionally involves identifying the mutagenized ENU region of interest via polymorphic markers. This traditional process has been fruitful in the past but requires fine mapping of the candidate region and exon-by-exon sequencing. This was slow, labour intensive and involved making assumptions about the underlying genetic cause of the observed phenotype. With the advancement of next generation sequencing (NGS), whole exome or genome sequence can be produced in a matter of weeks rather than years and new analysis techniques based on this data are rapidly reducing mutation identification time and increasing mutation characterisation analysis. Here, we explore the current and innovative strategies used to identify ENU mutations via NGS, their correlation to human disease and its impact on mouse genetics.

## Next generation sequencing

### Whole genome versus whole exome sequencing

There are many different NGS platforms ranging from those generating billions of short sequence reads of ~100 bp (Illumina), to those generating reads of >1000 bp, to those sequencing a single molecule. The comparison of these technologies is covered in other reviews (Quail et al. 2012; Mardis 2013). Early application of NGS undertook a ‘targeting’ approach where candidate regions resulting from positional mapping would be deep-sequenced in order to find the causative ENU lesion (Kurapati et al. 2012). Due to the reduction in sequencing cost, whole exome and whole genome approaches are becoming a mainstay for discovering novel mutations in mouse or human populations.

Whole exome sequencing (WES) typically refers to sequencing every protein-coding exon in the genome. It may also be extended to user-specific loci and non-coding regions including; micro-RNAs, lincRNAs, etc. DNA libraries containing targeted exons from genes are usually governed by gene sets from reputable resources such as the consensus coding sequence (CCDS) database and the RefSeq database (Pruitt et al. 2009, 2014). As the exome represents approximately 1.5 % of the genome (Lander et al. 2001), significantly higher sequence coverage can be achieved with WES compared to whole genome sequencing (WGS). For example, ~90 Gb of sequence data is required to achieve a 30× average coverage of the whole genome whereas only 3 Gb of sequence data is required for a 75× average coverage of the whole exome (Voelkerding et al. 2009; Bainbridge et al. 2010). Deeper sequence coverage is a clear advantage of exome sequencing as sequence depth is directly correlated with the sequence quality of a single nucleotide variation (SNV). However, coverage is more uneven with WES than WGS due to biases in targeted capture, hence higher mean coverage depths are required to detect coding variants and some regions remain consistently difficult to capture (Sims et al. 2014). For example, a recent study comparing the human Gencode annotation with current exon arrays found 5594 genes missing from the array geneset and inaccessible to WES (Coffey et al. 2011). NGS technologies have higher error rates than Sanger Sequencing, leading to increased false positives in mutation detection (Kircher and Kelso 2010; Ledergerber and Dessimoz 2011). This is somewhat offset when sequencing depth is increased; however, systematic biases will persist. Large-scale initiatives using WES to detect spontaneous mouse mutations and ENU-induced mutations have shown a good success rate (~40–75 %) for novel mutation detection (Boles et al. 2009; Fairfield et al. 2011). However, WES is reliant on gene annotations from databases that will not contain undiscovered exons or regulatory sequences such as enhancers or promoters, areas increasingly recognised as important in disease. Moreover, larger sequence variations such as structural variations (e.g. large insertions, deletions or translocations, etc.) that span exon boundaries will remain undetected. Previous ENU studies detected the majority of ENU-induced mutations in coding exons (Nolan et al. 2000; Quwailid et al. 2004); therefore, there is a preference for deeper sequencing using exome sequencing. There is likely to be an ascertainment bias in the past ENU literature due to difficulty in identifying non-coding variants (e.g. found in repetitious regions with limited functional annotation). However, interpretation of these regions is becoming a more tractable problem with resources to predict function in non-coding regions (Stamatoyannopoulos 2012) and WGS will make it easier to detect these mutations.

### General NGS pipeline

Sequence analysis to discover ENU mutations requires three basic steps: (i) alignment to a reference genome, (ii) variant detection and (iii) variant annotation. This pipeline usually occurs in an automated manner prior or in tandem with the isolation of the ENU causative mutation. This review will mostly concentrate on the specific detection of novel or ENU-induced mutations alongside characterisation as part of the second and third step. Briefly, mouse mutant sequence data are usually aligned to the reference (mm10) using a popular aligner (e.g. BWA, Maq). The alignment is the foundation for accurate mutation detection and is critical to identifying all possible variants. Currently a good alignment maps ~98 % of the reads with default parameters (e.g. usually two mismatches in the seed sequence). There are a plethora of widely used variant callers, including SAMtools (Li et al. 2009), Unified Genotyper in the Genome Analysis Toolkit (GATK)(DePristo et al. 2011), Platypus (Rimmer et al. 2014), etc. Typically variant calling involves two steps: genotype assessment and variant identification, both steps vary between different callers. Even though many variants will be common between the different callers, mutation detection should be carried out with one or more mutation detection tools to minimise false positives. There are many reviews on the different types of variant callers (Liu et al. 2013; Pirooznia et al. 2014). Lastly, annotating sequencing variants in terms of genomic position, functional context and potential clinical impact has become an essential part of sequence variant analysis. ENU NGS pipelines typically determine the genomic annotation of a SNV; intronic, exonic, missense, nonsense, splice site, regulatory region, etc. Three popular tools for variant annotation are ANNOVAR (Wang et al. 2010), NGS-SNP (Grant et al. 2011) and Variant Effect Predictor (McLaren et al. 2010). The impact of a sequence variant on the genome and phenotype is briefly discussed below. To our knowledge, relating a sequence variant directly to the phenotype is not yet standardised and would be challenge to the bioinformatic field.

As NGS technologies and detection of novel mutations in ENU-induced mice become commonplace, the requirement to streamline the mutation detection process to ensure cost efficiency has increased. Different mouse breeding schemes and the mutation detection methods developed are discussed below.

### ENU breeding and background

A variety of strains have been used, in a range of phenotype-driven screens, which have been reviewed in detail elsewhere (Acevedo-Arozena et al. 2008; Andrews et al. 2012; Wang et al. 2015). The most commonly used background is C57BL6/J, because this strain retains fertility at higher doses of ENU (Justice et al. 2000) and the number of mutations induced is proportional to the dose of ENU (Russell et al. 1982). A variety of breeding strategies can be employed reviewed below and in Acevedo-Arozena et al. 2008. Firstly, the simple outcross scheme, which enables the rapid identification of a map location; and secondly the inbred scheme, which relies on sequencing to map mutations, increasing the number of mutations present in G_3_ mice by breeding from two G_0_ mice. The main advantage of carrying out phenotypic screens on an inbred background is reduced variation in the data produced. Differences between strains in certain phenotypes result in greater variation in the baseline data, making detection of subtle phenotypes on a mixed genetic background more difficult and often requiring more mice to confirm a phenotype. For example, there is a significantly lower bone mineral density in C57BL/6J mice when compared to most other strains (Simon et al. 2013). This variance can however lead to the identification of phenotypic modifiers which may or may not be advantageous to the screen. Additionally certain inbred strains may be employed because of their susceptibility or resistance to certain phenotypes (Jonczyk et al. 2014; Banks et al. 2015).

A variety of breeding strategies have been utilised to maximise the number of mutations in the progeny that undergo screening. As long as a phenotype is detectable, and is amenable to relatively high-throughput screening, forward genetic screens can be used as a discovery platform to identify genes and pathways associated with a disease or pathway. A wide range of screens have been applied; from developmental processes, ex vivo and in vivo analysis of immune function (Andrews et al. 2012; Wang et al. 2015), through basic physiological functions (Hrabe de Angelis et al. 2000; Acevedo-Arozena et al. 2008) to more complex behavioural phenotypes (Nolan et al. 2000). Challenges can be applied to mouse phenotyping pipelines to discover novel gene function and screens have revealed modifiers of phenotypes or indeed disease progression (Vinuesa and Goodnow 2004; Buchovecky et al. 2013).

Coupled with the increased efforts of the more sophisticated phenotyping pipelines (Brown and Moore 2012) are the new and innovative ways to detect mutations using NGS, ranging from large structural variants to small insertions and deletions (indels) to single nucleotide polymorphisms (SNPs). ENU mutations are typically SNVs and to a lesser extent, small indels. Since the emergence of NGS there has been an evolution of ENU mutation detection strategies, making ENU an efficient and attractive method to generate mouse models of human disease (Andrews et al. 2012; Potter et al. 2015).

### Methods for mutation mapping and detection

#### Method 1: candidate region approach

**Fig. 1:**
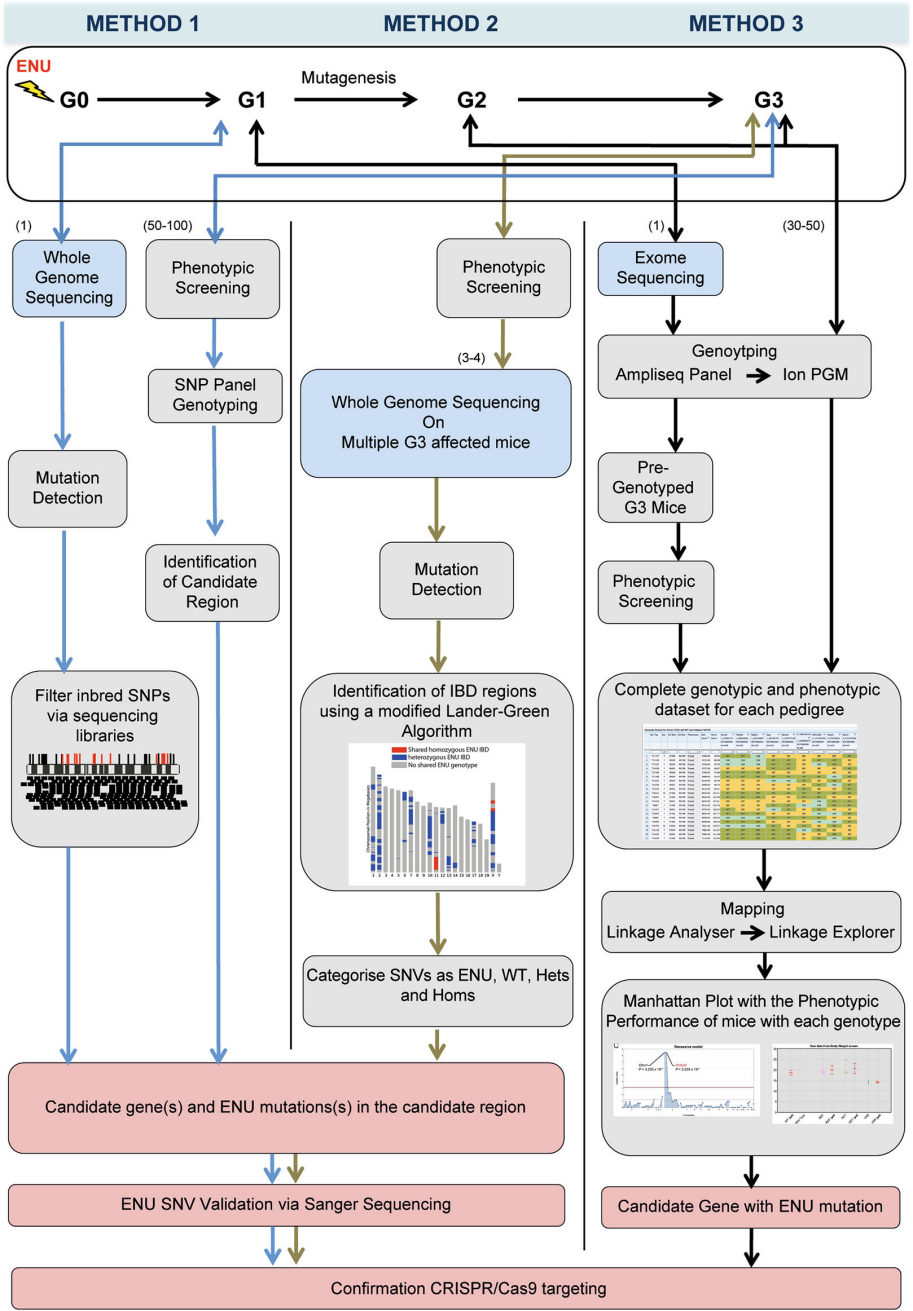
Overview of ENU mutation detection methods used on DNA-Seq data. *Method 1* Male C57BL/6J mice mutagenized with ENU are bred to produce 50–100 third generation (G_3_) mice carrying mutations mostly in the heterozygous state. The G_1_ male founder of each pedigree is sent for whole genome sequencing. The G_3_ mice are put through a phenotyping screen and affected mice are genotyped with a SNP panel to identify ENU regions. Specific ENU SNPs within the candidate region are validated via Sanger Sequencing. After secondary phenotyping and inheritance testing a copy of the potential causative mutation may be generated with CRISPR/Cas9 targeting. *Method 2* Two C57BL/6J mice are mutageneised with ENU, each are paired with WT C57BL/6J females to produce third generation mice carrying 4 possible haplotypes, ENU1, ENU2, WT1 and WT2. After phenotype testing 3 phenovariant G_3_ mice are sent for low coverage whole genome sequencing. Shared homozygous ENU variants seen in all 3 mice cluster in an IBD region, detected using the Lander-Green algorithm. Coding variants within the IBD are validated via Sanger Sequencing. Alternative alleles may be generated using CRISPR/Cas9 targeting. *Method 3* Male C57BL/6J mice mutagenized with ENU are bred to produce 30–50 third generation (G_3_) mice carrying mutations in homozygous and heterozygous state. The G_1_ male founder of each pedigree is subjected to exome sequencing, and data are used to generate Ampliseq panel primers for amplification of mutated loci from G_2_ and G_3_ mouse DNA, followed by Ion PGM 200-bp sequencing. Genotyping data are uploaded to Mutagenetix prior to phenotypic screening. Quantitative phenotype data are entered into Mutagenetix and used with genotype data for mapping by Linkage Analyzer. Calculated *P* values for non-linkage, Manhattan plots, and scatter plots of phenotypic data for every mutant allele are displayed by Linkage Explorer. Confirmation of candidate genes depends on duplication of the mutant phenotype by a second allele, which may be generated by CRISPR/Cas9 targeting

**Fig. 2:**
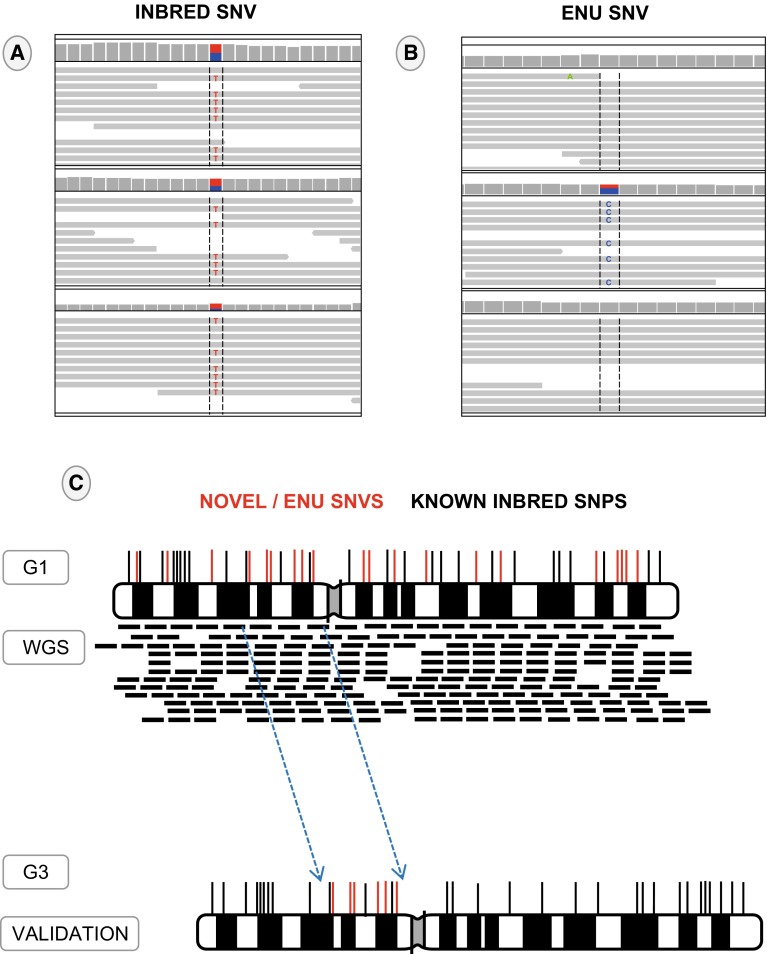
Identification of ENU mutations using polymorphic markers on a mixed background. **a** WGS of 3 G_1_ samples showing heterozygous inbred SNP sites, which are shared among all samples. These sites are eliminated from the ENU mutation list; the remaining SNPs (**b**) are novel or ENU-induced. **c** Illustrates a simplistic view of randomly distributed ENU SNVs in a chromosome of a G_1_ mouse. The WGS of the G_1_ denotes the genomic location of the ENU SNVs in the candidate region of an affected G_3_ mouse

Whilst several phenotype-driven ENU screens have been run or are still underway, to our knowledge, the Harwell Ageing Screen is the first to apply whole genome sequencing in a high-throughput, unbiased approach to discover genetic lesions that result in a detectable phenotype. The two mouse strains that are used by MRC Harwell to generate mutant mouse lines are C57BL/6J and C3H/HeH. Initially, male mice are injected intraperitoneally with ENU doses of 1 × 120 mg/kg, and then 2 × 100 mg/kg with a week between each dose. These mutagenised male mice (G_0_) are then mated with wild type females to give mice that are heterozygous for every ENU-induced mutation (G_1_). These can be subjected to phenotype-driven screening programs, with the intent of discovering dominant mutations, or further breeding can be carried out to generate homozygous mutant mice (G_3_) to identify recessive mutations resulting in phenotypes. The Harwell Ageing Screen has opted to sequence the G_1_ mouse in order to detect all of the ENU-induced ENU mutations contained within a pedigree. In parallel to G_1_ sequencing, G_3_ phenotyping is carried out. Once a phenotype of interest is identified (e.g. >3 mice are phenodeviant at any one timepoint) the affected G_3_ mice undergo positional mapping. Positional mapping aims to identify the recombinant mapping region(s) containing the causative ENU lesion (Fig. 1). Typically the breeding scheme will include a highly polymorphic background strain to provide polymorphic genetic markers flanking the ENU lesion. The interval size is characterised by the density of polymorphic markers alongside the number of recombination events. Figure 2 shows an ENU region in the genome flanked with polymorphic markers. Once the candidate region in the G_3_s is narrowed to a manageable size (this can be anything ranging from ~30 Mb to the whole chromosome), all coding and non-coding variants in the respective G_1_ loci are identified in the WGS mutation detection pipeline. The NGS and mutation detection pipeline used at Harwell involves mapping sequence reads to the mouse reference (currently mm10) and calling SNVs using an established SNV caller such as GATK or SAMTOOLs. Subsequent prioritisation of the variants occurs (discussed below) and the G_3_s are genotyped for the chosen variants to confirm inheritance of the putative causative mutation. This ‘drill down’ approach allows for the rapid discovery of multiple causative ENU mutations in a pedigree when only sequencing one mouse, whilst also generating a library of potentially functional mutations available for a gene-driven approach in the G_1_ archive (Quwailid et al. 2004). The main challenge of mutation detection is distinguishing genuine ENU lesions from the background noise resulting from nucleotide errors in the sequence reads. Over the years a number of typical steps have been employed to remove the false positives. These steps include one or more of the following: a read depth threshold where variants found in less than the allotted number of reads are ignored, a quality threshold where variants in poorly mapped reads are ignored and inbred SNP identification where variants overlapping background SNV sites are ignored (Simon et al. 2012). This prioritisation and filtering of SNVs is a crucial step in the NGS pipeline as false discovery of erroneous SNVs masquerading as real ENU variants can result in incorrect candidate genes, whereas over-filtering can result in the exclusion of the real causal mutation, resulting in the failure of the experiment.

To date, Harwell has used this NGS pipeline and mutation detection strategy on >70 mouse genomes including 44 genomes, both G_3_ and G_1_ for the Harwell Ageing Screen. Harwell found coding ENU mutations (missense, splice and nonsense) in the candidate ENU regions of 41 of the 44 genomes. Further characterisations of these mutations are underway including inheritance testing, secondary phenotype testing and molecular examinations.

#### Method 2: rapid causative mutation finding without use of an outcross

Method 1 represents an early adoption of NGS for ENU mutation detection which relied on outcrossing and coarse mapping (Arnold et al. 2011; Fairfield et al. 2011; Leshchiner et al. 2012; Sun et al. 2012). A more efficient method to rapidly isolate causative ENU mutations should avoid outcrossing, be quick and cost effective, reliable and comprehensive.

**Fig. 3:**
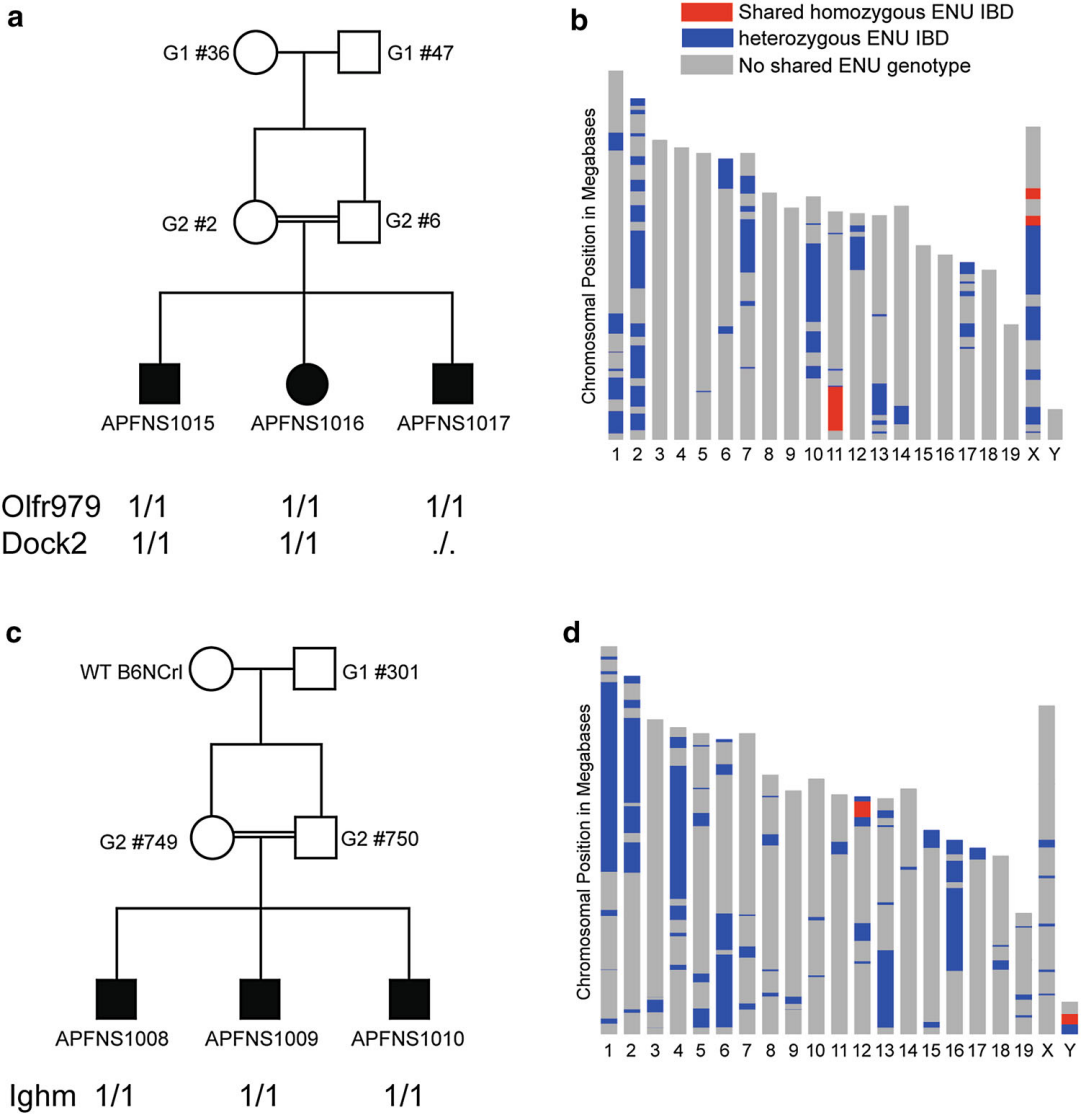
Identification of IBD regions using a modified Lander–Green Algorithm, **a** pedigree in strain APFN1015-1017, the sequenced mice are* shaded*. The gene and genotype for the candidate mutation is shown for each sequenced individual. 1/1 indicates homozygous for mutation, *./.* indicates insufficient coverage to call the genotype at that locus in an individual. **b** Plot showing IBD homozygous (*red*) and IBD heterozygous (*blue*) regions predicted by the Lander–Green-based algorithm in APFN1015-1017. **c** Pedigree for strain ENU22 with genotypes for the Ighm mutation. **d** Plot showing IBD regions for ENU22

Bull et al. published the first method to eliminate outcrossing to a second inbred strain or additional breeding steps after G_3_, using an identity by descent (IBD)-based approach that infers shared genomic intervals across mice within a pedigree and simultaneously isolates causative ENU mutations (Bull et al. 2013). The method is based on low coverage whole genome sequencing of multiple phenotypically affected mice, and an implementation of the Lander–Green algorithm (Rabiner 1989). The algorithm harnesses knowledge of the pedigree structure to infer the inheritance of founder genotypes. In contrast, methods that simply search for shared mutations will pick up false positives due to shared sequencing errors. They found that excluding shared variants outside of shared genomic intervals removes 75 % of putative shared mutations. Further modelling and empirical data shows that one or two candidate causative ENU mutations can be isolated based on sequencing 3 G_3_ mice for a recessive trait or 6 G_3_s for a dominant trait (Fig. 3).

Fine mapping of regions inherited from an ENU ancestor is achieved based on the density of variation, despite the scarcity of ENU variants across the inbred C57B6 genome, using whole genome rather than whole exome sequencing. The depth of coverage in shared genomic intervals is the sum of the depth across all sequenced mice, and the method uses local genotype context to isolate a causative mutation. Therefore, the actual coverage depth per mouse can be very low; in this method all affected individuals from a pedigree are sequenced on one lane of an Illumina Hiseq machine; achieving 12–15 fold combined coverage across the causative variant locus. Bull et al. found this was sufficient to reliably call a homozygous or heterozygous point mutation, since WGS has less variability in depth of coverage than WES (Sims et al. 2014).

The current technique applies WGS to affected G_3_ individuals within a pedigree; therefore, the delay between identifying a phenotype of interest and isolating the mutation is the sum of the time to run the sequencing (typically 1–2 weeks), the time ‘queuing’ for a sequencing run, which varies between institutions plus the time to run the NGS pipeline. Whilst this is a significant improvement over earlier methods that relied on outcrossing and further breeding beyond G_3_ for mapping, an approach that generates genotyping data in parallel with phenotyping pipelines, as described by the Beutler group below, avoids this delay altogether. As the costs of WGS continue to fall, it will become feasible to apply WGS to all mice within the pedigree in parallel to phenotyping, rapidly generating a rich database linking phenotype and genotype across coding and non-coding regions.

#### Method 3: real time identification of ENU-induced mutations in mice

The above methods use massively parallel sequencing of whole mouse genomes or exomes and have arguably exposed genetic mapping as the rate-limiting step in forward genetics. Most ENU-induced mutations are easily found (Andrews et al. 2012); however, finding the causative mutation has remained a time-consuming task. Light sequencing of bar-coded samples from G_3_ mice for the purpose of genotyping remains a fairly costly proposition, and is usually applied *post facto* only to pedigrees that display a phenotype (Bull et al. 2013). This means that finding causative mutations is not truly a real-time process, and also precludes the systematic exoneration of non-causative mutations from the screen as a whole.

The Beutler lab developed an alternative approach that permits declaration of causative mutations concurrent with phenotypic screening (Wang et al. 2015), without a requirement for outcrossing and backcrossing or intercrossing as practiced in mapping based on meiotic recombination. Their approach combines exome sequencing and high-throughput genotyping to determine zygosity at all mutation sites in all G_3_ mice before phenotypic data are acquired, and uses automated computational mapping to assign causality in real time (for overview see Fig. 1). Mice are bred to produce 30–50 G_3_ mice per pedigree, a number sufficient to detect concordance between traits of moderate strength and homozygosity at a particular locus, assuming a neutral effect on viability. A single G_1_ male serves as the founder for each pedigree, and is subjected to whole exome sequencing to identify all possible mutations transmitted to G_3_ mice. Prior to phenotypic screening, the zygosity of these mutations is determined by genotyping G_2_ and G_3_ mice and data are uploaded to the Mutagenetix database to await linkage analysis together with phenotypic data. All 30–50 G_3_ mice are screened in a single experiment on the same day; with the exception of visible phenotypes (affecting, for example, coat colour or behaviour), phenotypic data are quantitative in nature.

Automated linkage analysis is performed by two software programs; Linkage Analyzer and Linkage Explorer, they are based on classical principles of genetic mapping. That is, correlation is determined between genotypes at mutated loci and the presence or absence of a qualitative phenotype, or the magnitude of a quantitative phenotype, with reference to recessive, additive (semi-dominant), or dominant models of inheritance. This determination is made for each mutation site in all mice in a pedigree. The assessment of linkage depends on the probability of association between genotype and phenotype as calculated using a likelihood ratio test from a linear regression model (Wang et al. 2015). With this method, phenovariance is ascertained computationally, thereby eliminating the need for the researcher to designate mice as affected or non-affected.

Linkage Analyzer, the core mapping program, calculates probabilities of association between genotype and phenotype for every mutation subjected to every screen using recessive, additive and dominant transmission models. It detects associations with quantitative and qualitative traits and with lethal effects when homozygosity is significantly under-represented among G_3_ mice in a pedigree. Additionally, the program identifies complex linkage for phenotypes that depend on two unlinked mutations in any combination of zygosities. Over time, multiple variant alleles of most genes are tested phenotypically, and Linkage Analyzer can combine pedigrees with identical or non-identical allelic mutations to make “superpedigrees.” These are analysed as single pedigrees for genotype**–**phenotype associations including linkage to lethality.

**Fig. 4:**
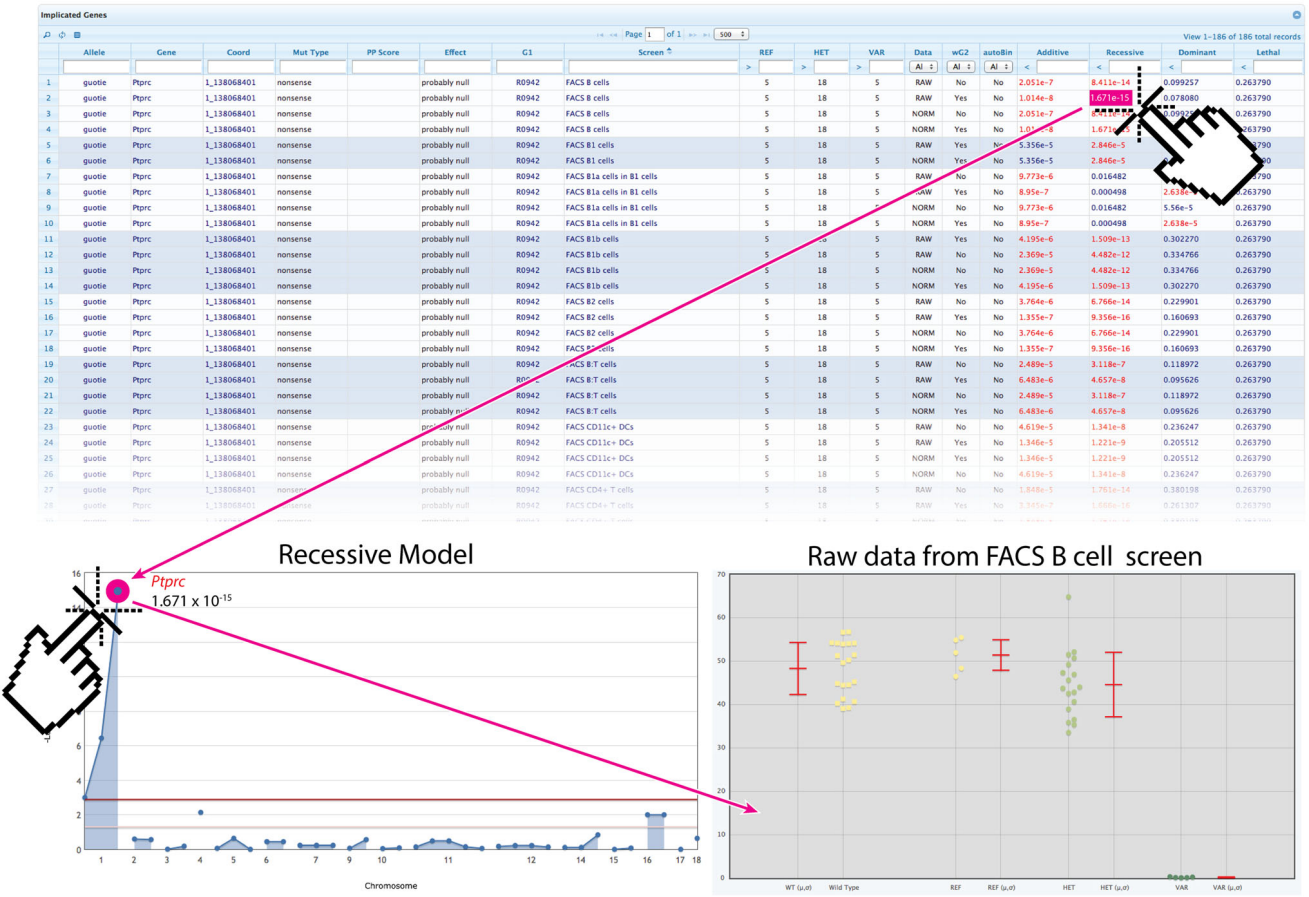
Presentation of mapping data by Linkage Explorer. A portion of a typical results table (*top*) displays *P* values for all three transmission models for each mutation, here sorted by phenotype. *P* values are linked directly to the Manhattan plot (*lower left*), where mousing over data points reveals the gene name and associated *P* value. Clicking a data point opens the scatter plot of phenotypic data graphed versus genotype (REF, homozygous for wild type allele; HET, heterozygous for mutant allele; or VAR, homozygous for mutant allele) for the mutation in question (*lower right*). *μ* mean, *σ* standard deviation

*P* values for non-linkage calculated by Linkage Analyzer are tabulated and presented by Linkage Explorer in an online format with one-click access to Manhattan plots for each phenotype and inheritance mode, and from there direct links lead to scatter plots of phenotypic data graphed versus genotype for every variant allele (Fig. 4). A key feature of Linkage Explorer is the ability to narrow or expand the list of positive associations by varying the stringency of criteria for linkage, and by targeting analyses to specific genes, phenotypes, pedigrees and mutation types or effects (Table 1). The nature of each mutation, PolyPhen-2 score, and its effect at the protein and gene levels are also accessed with a single click in Linkage Explorer.

**Table 1:** Parameters that may be specified in linkage explorer

Parameter	Notes
*Single or double locus analysis*	
Gene	Will return all phenotypes linked to mutations of the specified gene(s), along with associated *P* values
Phenotypic screen	When specified, will return mutations linked to the phenotype(s) tested in the specified screen(s)
Pedigree or mouse/mice	Will return all genotype–phenotype associations identified in the specified pedigree or the pedigree of which the specified mouse (mice) is (are) part, along with associated *P* values. Named according to eartag of G1 male founder
Total mouse numbers	Will restrict linkage analysis to pedigrees containing a specified range or number of G_3_ mice
Allele name (phenotype)	Will return all mutations linked to the specified phenotype, along with associated *P* values
Mutation type	Will restrict linkage analysis to the specified mutation type(s): nonsense, missense, makesense, critical splicing, noncritical splicing
Predicted effect of mutation	Will restrict linkage analysis to the specified mutation effect: probably null (corresponds to nonsense and critical splicing mutations); or probably damaging, possibly damaging, probably benign as determined by PolyPhen-2
*P* value cutoff	Will display genotype–phenotype associations with *P* (non-linkage) ≤ the value specified; Bonferroni correction may be applied
Minimum number of HET or VAR mice screened	Will return genotype–phenotype associations tested with at least the specified number of HET (heterozygous) or VAR (homozygous mutant) mice
‘Raw + Norm’ switch	When applied, enforces *P* value cutoff for both raw and normalized datasets. Otherwise, enforces *P* value cutoff for either raw or normalized datasets
Direction of phenovariance	Quantitative phenotype scores either higher than or lower than wild type scores
Number of linkage peaks	Will return genotype–phenotype associations for which a specified number of linkage peaks exceed the specified −log_10_[*P*(non-linkage)] in the Manhattan plot for recessive, dominant or additive models of linkage. This parameter is useful for filtering results to show only strong, unambiguous genotype–phenotype associations
Date of data collection	

The speed of mapping by Linkage Analyzer now exceeds the rate of production and screening of G_3_ mice, and linkage assignment occurs within minutes of the entry of phenotypic data to the database. There are several other advantages to this approach. Mapping of quantitative low penetrance and weak phenotypes, which may be difficult to assign to affected vs. non-affected groups, is made possible by the statistical determination of phenovariance and by superpedigree analysis, which increases the power to detect linkage by enlarging the mapping population. Complex traits dependent on two loci can be solved in pedigrees of sufficient size. Moreover, because all mutations in a pedigree are known, not only causative mutations but non-causative mutations (constrained by a specified *P* value) can be declared. This approach also permits the measurement of saturation, with an upper limit set by the number of genes tested in homozygous state with “probably damaging” missense or null alleles, and a lower limit set by the number of genes with null alleles. As for other mapping strategies described in this review, the limitations of exome capture and massively parallel sequencing apply to our approach. In addition, although the majority of ENU-induced phenotypes have been shown to arise from mutations in coding sequence (Fairfield et al. 2011; Arnold et al. 2012), it remains possible that causative intronic mutations would on rare occasions be missed or attributed to closely linked exonic mutations. Routine CRISPR/Cas9 targeting of implicated genes is therefore used to confirm mapping data.

To date, the Beutler lab has used Linkage Analyzer and Linkage Explorer to test a total of 53,966 mutations in 16,350 genes for their ability to cause phenovariance in 135 screens of immunological function. The mutations were distributed within 22,421 G_3_ mice from 876 pedigrees. Linkage Analyzer is freely available for download and online data analysis of selected pedigrees via the Mutagenetix website (https://mutagenetix.utsouthwestern.edu/linkage_analysis/linkage_analysis.cfm).

## Mutation annotation and consequence

Sequence variation validation typically involves four steps: (i) confirmation of linkage by genotyping, (ii) secondary phenotyping, (iii) cloning the mutation and (iv) producing an alternate allele to confirm the causative allele. With the information generated by NGS, the confirmation of phenotype association with a novel gene is not such a stringent requirement for the confirmation of association, as there is little doubt over whether a second, unidentified allele is associated with a particular phenotype, as was the case with candidate gene sequencing strategies. Furthermore, the advent of CRISP/Cas9 technologies and the easy availability of KO lines (Koscielny et al. 2014) is a great boon to confirmation of a functional link between a novel allele or gene and a phenotype. Alongside ENU validation is usually the in silico examination of the mutation consequence, its influence on the phenotype and association to human disease. ENU-induced mutations provide a full range of alleles including null (loss of function), hypomorphic (reduced function), hypermorphic (gain of function) and neomorphic (novel function); and better model the genetic variation found in the human genome. Moreover, these mutations can reveal gene functions that would not have been discovered through the analysis of null alleles alone (Qian et al. 2011). The coding causative variants are usually classified based on their functional consequence to the genomic sequence; namely missense, nonsense, synonymous and splice site mutations. Nonsense and splice site disruptive SNVs are thought to cause loss of function mutations, while missense mutations can be damaging or tolerant to the protein structure and function (Khurana et al. 2013). The current major challenge in analysing genetic variants is in interpreting the functional affect a mutation has on the gene and/or genome.

A variety of methods are available online to predict the functional effects of SNVs. These methods can be classified into different categories, based on the algorithms implemented for prediction (Table 2). Multiple sequence alignment-based tools implement information on amino acid conservation among homolog protein sequences at particular loci (Ng and Henikoff 2003; Reva et al. 2011). Other tools implement sequence data alongside three-dimensional structure to predict the functional impact of the amino acid on the protein. Tools which combine functional annotation alongside structural data arguably give the best indication of severity. For example, Mutation Taster combines information from different data sources including evolutionary conservation, splice site changes and expression data and PolyPhen2 uses a naïve Bayes classifier which implements eleven features, of which eight are sequence-based while three are structure-based (Adzhubei et al. 2010; Schwarz et al. 2014). Currently there are 4897 solved distinct protein structures, a limiting factor when assessing mutational consequence; therefore, most predictions involve only a local structure alignment. As protein structure information increases the accuracy of SNV functional predictions will also increase. This information will not only impact the SNV role in protein structure but also the mutation’s role in protein–protein interactions and post-translational modifications (Ren et al. 2010; Wendl et al. 2011; De Baets et al. 2012). In some cases, information on the SNV-containing protein domain alongside prior knowledge of protein–protein interactions will be sufficient to determine some affects the mutation has on the pathology of disease.

**Table 2:** Tools used to predict the functional or structural impact of SNVs

Tool	URL	Notes	Organism	Reference
*Conservation*				
SiFT	http://sift.jcvi.org/	Predicts effect of SNVs	Human and known mouse SNPs (dbSNP)	(Kumar et al. 2009)
MutationAssessor	http://mutationassessor.org	Predicts effect of SNVs	Human data: cancer studies	(Reva et al. 2011)
Provean	http://provean.jcvi.org/	Predicts effect of SNVs, insertions and deletions	Organism independent	(Choi et al. 2012)
*Structure*				
SNPs3D	http://www.snps3d.org/	Predictions based on sequence, 3-D structure, biological networks	Human, useful for association studies	(Yue et al. 2006)
*Machine learning/multiple datasets*				
Polyphen-2	http://genetics.bwh.harvard.edu/pph2/	Implements MSA, amino acid changes, evolutionary conservation, SNV site hypermutability. Uses a naïve Bayes classifier	Human, can be adapted for mouse genome (standalone)	(Adzhubei et al. 2010)
MutationTaster2	http://www.mutationtaster.org/	Machine learning on evolutionary conservation, splice site changes, gene expression and protein features. Uses a Bayes classifier	Human, uses 1000G data	(Schwarz et al. 2014)
SNAP	https://www.rostlab.org/services/snap/	Uses neural networks for evolutionary conservation, secondary structure, solvent accessibility	Human	(Bromberg and Rost 2007)
Site Directed Mutator (SDM)	http://mordred.bioc.cam.ac.uk/~sdm/sdm.php	Uses a potential free energy function for protein stability; algorithm uses environment-specific substitution tables to calculate stability, predicts disease association	Organism independent	(Worth et al. 2011)
*Post-translational modifications*				
PhosSNP	http://phossnp.biocuckoo.org/	Predicts SNV effect on PTM	Human	(Ren et al. 2010)
SNPeffect	http://snpeffect.switchlab.org/	Predicts SNV effect on PTM, structural features of proteins, subcellular localization and interactions	Human	(De Baets et al. 2012)
*Protein–protein interactions*				
MuSiC	http://gmt.genome.wustl.edu/packages/genome-music/	Predicts SNV effect on pathways (Cancer studies). To segregate passenger mutations from truly significant mutations	Human	(Dees et al. 2012)

*MSA* multiple sequence alignment, *PTM* post-translational modifications

The success of phenotype-driven screens in detecting mutants that inform us about biological function is not in doubt but to date, the vast majority of such mutations that have been detected affect coding regions, with a minority being identified as occurring in non-coding regions (Lewis et al. 1991; Masuya et al. 2007). This, it could be argued, is due to a sampling bias as only coding and splice regions have been examined in the majority of programmes who employed a candidate gene approach or NGS technologies (Quwailid et al. 2004; Acevedo-Arozena et al. 2008; Andrews et al. 2012; Wang et al. 2015). The debate on the functional contribution of non-coding DNA continues (Consortium 2012; Eddy 2012; Doolittle 2013) but MRC Harwell’s data presents one of the first unbiased high-throughput examination of the link between phenotype and genotype on a stable genetic background in a mammalian physiology thus enabling us to begin to explore the contribution of non-coding DNA to phenotype. Despite the majority (~97.5 %) of randomly induced mutations being detected in non-coding regions, the overwhelming majority of phenotypes identified (41/44) can be assigned to protein changes. This does seem to suggest that the majority of ‘function’, where changing the sequence results in a detectable phenotypic change, is associated with the gene. However, there are caveats; the phenotypic interrogation of the mutant pipeline of mice is not exhaustive and cannot detect every possible phenotype. It is, however, an unbiased approach as the phenotypes detected undergoes mapping and then sequencing with no assumption of the underlying genetic lesion. It may be that non-coding DNA is more tolerant of sequence changes and is thus under-represented. As more phenotyping and whole genome sequencing is undertaken we will provide further information about the links between sequence and phenotype, particularly concerning the contribution of non-coding DNA to phenotype but these initial results provide a tantalizing glimpse into the functional analysis of DNA and seems to fit with current hypotheses (Palazzo and Gregory 2014). These results will have a significant impact on the search for causative alleles using deep sequencing of patients, suggesting that the current technique of primarily using next generation sequencing will indeed find the majority of causative alleles.

## Human correlation

A key goal in understanding human disease and gene dysregulation is to discover and interpret all the genetic variations that can occur in the human population. Advances in sequencing technology and related tools have made it feasible to sequence many human genomes and catalogue all the possible variations. The 1000 Genomes Project, started in 2008, aimed to identify 95 % of the variants that occur in ~1 % of the population and evaluate the feasibility of large-scale sequencing to capture true variants or artefacts (Genomes Project et al. 2010). The project has provided a catalogue of low to high frequency variants which are already starting to support the development of genotyping products as well as a list of background variants to aid the identification of disease-causing and non-disease-causing variants. In parallel, GWAS has become a valuable tool for discovering common variants linked to disease. It is becoming clear that GWAS and other human studies will have considerable effect on human health, especially as independent studies are starting to report the same genes or variants associated with particular diseases (Abad-Grau et al. 2012). GWAS is increasing our understanding of the genetic etiologies underlying all types of diseases ranging from common to complex etiologies. Some reports imply some human diseases are not solely caused by a single variant but rather a combination of multiple common variants exerting a weak affect alongside more severe or stronger effect variants (Visscher et al. 2012). While others find human diseases are associated with multiple variants acting in unison where each variant lies within a single Mendelian disease-causing loci and has the potential to be deleterious in their own right (Blair et al. 2013). With the methods outlied above we have the opportunity with sequencing and advanced phenotyping strategies to correlate ENU mutations with human disease more effectively, rapidly and accurately. Key advantages of the phenotype-driven approach in mice are the number of mutations that can be induced, the range of phenotyping that can be carried out from birth, and the enhanced ability to discover novelty. Human-based studies still rely heavily on published data, and proving a novel function for a gene or the association of a novel gene with a particular phenotype is more difficult than in mouse studies where functional data are more easily obtained and inheritance can be demonstrated rapidly. Not only is this seen with the projects described above but also with other initiatives where mutation detection in NGS data may uncover novel disease-causing variants. For example, modifier screens, where sequencing of ENU mutants is used to discover novel genes that alter a phenotype (Rubio-Aliaga et al. 2007), highlight potential therapeutic targets and generate more complex models of disease. Partnerships between human and mouse geneticists where human-cohort studies run alongside sequencing mouse models with similar phenotypes (Tucci et al. 2014) and mouse GWAS-like studies where multiple mouse lines with varying phenotype severity are sequenced and genotyped to determine regions of linkage disequilibrium or QTLs could therefore be extremely beneficial. Only time will tell if human and mouse sequencing partnerships translate into a clinical setting, in the meantime such studies are continually advancing our understanding of the genetic contribution to disease and physiological processes.

## Conclusion

In the present review, we have outlined three disparate methods to detect ENU mutations in NGS data; all methods have been successful in finding an abundance of ENU causative mutations. It is possible a particular method is suited to a specific ENU study, for example, the traditional mutation detection method, method 1 may be employed when investigating a single ENU mouse on a mixed background as gross mapping of the candidate region is relatively easily achieved. Methods 2 and 3 take a population-based type approach with ENU where multiple samples are used to predict ENU mutation. Method 2 is an extension of method 1 and is more effective when the ENU mouse is on an inbred background. Method 3 automatically combines phenotype and genotype information in a GWAS-type fashion to generate linkage region containing the causative gene. As more ENU mutations are characterised the efficient use of CRISPR/Cas 9 genome editing system will become increasingly valuable as a way to validate the ENU mutations. In addition CrispR/Cas 9 can be used to mimic any human deleterious variation. The future of ENU may incorporate the combination of ENU and CRISPR/Cas 9 as this enables both the discovery novel genetic interactions alongside mimicking human disease variants.
